# Maternal characteristics and type of prenatal care associated with peregrination before childbirth

**DOI:** 10.11606/s1518-8787.2019053001087

**Published:** 2019-08-15

**Authors:** Rosemar Barbosa Mendes, José Marcos de Jesus Santos, Daniela Siqueira Prado, Rosana Queiroz Gurgel, Felipa Daiana Bezerra, Ricardo Queiroz Gurgel

**Affiliations:** I Universidade Federal de Sergipe Universidade Federal de Sergipe Departamento de Enfermagem São CristóvãoSE Brasil Universidade Federal de Sergipe. Departamento de Enfermagem. São Cristóvão, SE, Brasil; II Universidade de São Paulo Universidade de São Paulo Escola de Enfermagem de Ribeirão Preto Programa de Pós-Graduação Enfermagem em Saúde Pública Ribeirão PretoSP Brasil Universidade de São Paulo. Escola de Enfermagem de Ribeirão Preto. Programa de Pós-Graduação Enfermagem em Saúde Pública. Ribeirão Preto, SP, Brasil; III Universidade Federal de Sergipe Universidade Federal de Sergipe Departamento de Medicina São CristóvãoSE Brasil Universidade Federal de Sergipe. Departamento de Medicina. São Cristóvão, SE, Brasil; IV Universidade Tiradentes Universidade Tiradentes Departamento de Enfermagem AracajuSE Brasil Universidade Tiradentes. Departamento de Enfermagem. Aracaju, SE, Brasil; V Faculdade Estácio de Sergipe Faculdade Estácio de Sergipe Departamento de Enfermagem AracajuSE Brasil Faculdade Estácio de Sergipe. Departamento de Enfermagem. Aracaju, SE, Brasil

**Keywords:** Pregnant Women, Midwifery, Maternal-Child Health Services, supply & distribution, Health Services Accessibility, Equity in Access to Health Services

## Abstract

**OBJECTIVE:**

To analyze the maternal characteristics and type of prenatal care associated with peregrination before childbirth among pregnant women in a northeastern Brazilian state.

**METHODS:**

Quantitative and transversal study, with descriptive and analytical approaches, part of the *Nascer em Sergipe* research held between June 2015 and April 2016. A total of 768 puerperal women proportionally distributed across all maternities of the state (n = 11) were evaluated. Data were collected in interviews and from prenatal records. The associations between antepartum peregrination and the exposure variables were described in absolute and relative frequencies, crude and adjusted odds ratios and their respective confidence intervals.

**RESULTS:**

Antepartum peregrination was reported by 29.4% (n = 226) of the interviewees, most of whom sought care in a single service before the current one (87.6%; n = 198). It should be noted that antepartum peregrination was less frequent among women aged ≥ 20 years old (OR = 0.50; 95%CI 0.34–0.71), with high education level (OR = 0.42; 95%CI 0.31–0.59) and a paid job (adjusted OR = 0.59; 95%CI 0.41–0.82), who had been instructed during prenatal care about the referral maternity for childbirth (adjusted OR = 0.88; 95%CI 0.42–0.92), and who used the private service to receive prenatal (adjusted OR = 0.44; 95%CI 0.18–0.86) or childbirth (adjusted OR = 0.96; 95%CI 0.66–0.98) care. No statistical evidence of associations between gestational characteristics and the occurrence of peregrination was observed.

**CONCLUSIONS:**

Antepartum peregrination suffers interference from the mother’s socioeconomic characteristics, the type of prenatal care received and the source of funding for childbirth.

## INTRODUCTION

Antepartum peregrination refers to the pregnant woman’s search of hospitalization for labor in more than one health service, which significantly increases the risks of complications in parturition and even of maternal or fetal death^1,2^. The longer this search, the greater the distance traveled by the pregnant woman in labor and, usually, the lower the probability of adequacy of the services found to her needs, especially in cases of patients who have been previously classified as high clinical or obstetric risk during prenatal care^1^.

In Brazil, although Law 11.634/2007^3^ ensures the right of every pregnant woman to knowledge of and prior connection to the referral maternity where she will give birth, a nationwide study showed that it is not satisfactorily complied with in the country, since only 58.7% of the 23,940 women interviewed had been instructed on it. The percentage of antepartum peregrination found by these authors was 16.2%, the worst situation having been found in the Northeast region, where the percentage found was 25.1%^4^. It should also be noted that the most recent report by the World Health Organization (WHO) on the maternal mortality ratio (RMM) between 1990 and 2015 showed that Brazil did not reach the fifth goal of the millennium development goals (MDGS), which advocated a 75% reduction in the RMM in the same period^5,6^.

The main causes of antepartum peregrination are related to the deficiencies of prenatal care and to the low number of obstetric beds available in normal-risk maternities. Other reasons are the lack of medical professionals and inputs and equipment in these services. These situations reinforce the importance of effective communication between primary care professionals and those who work in low, medium and high-complexity maternities, thus enabling an adequate functioning of these parturients’ referral and counter-referral system^4,7^. Albuquerque et al.^7^ and Souza et al.^8^ believe that the scarcity of national publications on antepartum peregrination is also one of the main unfavorable factors for minimizing or even solving this problem, being motivated mainly by the difficulty in obtaining quantitative information about its frequency among Brazilian women, since there are no public databases for this purpose in the country.

Thus, when considering that the immediate care of pregnant women in labor is essential to reduce maternal/fetal deaths and that the study of factors associated with late hospitalization may support the development of measures to prevent peregrination, the objective of this study was to analyze the maternal characteristics and the type of prenatal and childbirth care associated with antepartum peregrination among pregnant women in a northeastern Brazilian state.

## METHODS

This is a quantitative and transversal study, with descriptive and analytical approaches, part of the *Nascer em Sergipe* research held between June 2015 and April 2016. A total of 768 puerperal women proportionally distributed across all public, private and mixed maternities of the state (n = 11) were evaluated. The method used in the *Nascer no Brasil*^9^research was reproduced, with training of the local team by the researchers of Fundação Oswaldo Cruz (FIOCRUZ) who coordinated the national study.

The sample size calculation, with a 95% confidence level, had a probabilistic design in two stages. The first corresponded to the health institutions and the second to the puerperal women. All maternities of the state that registered at least 500 deliveries in 2012 were eligible, resulting in seven institutions in the countryside and four in the capital, five public, four mixed and two private. The puerperal women were selected by simple random sampling from a daily hospitalization list, all females with live fetus delivery and dead fetus delivery with birth weight ≥ 500 g or gestational age ≥ 22 weeks having been considered eligible. Women who did not speak or understood the language (Portuguese) and who had serious mental disorders were not eligible for the study. The allocation adopted for the sample number’s distribution was proportional to the institution’s size (n = 768).

The interviewers remained at least seven days in each institution. If the number of puerperal women could be reached before this period, a number was randomly drawn to serve as limitation of the daily number of interviews, so that the seven days were reached. In-person interviews with the puerperal women were conducted with a minimum interval of 6 hours after delivery and data were extracted from their medical records after discharge. The prenatal records were photographed and the information was entered in the database. More detailed information on data collection may also be found in Leal et al.^10^

The descriptive results of the puerperal women’s answers to questions related to antepartum peregrination are presented below: 1. “Did you receive prenatal care during this pregnancy?”, 1.1. “During prenatal care, were you instructed about the referral maternity for childbirth?”, 2. “Before being admitted to this institution, believing to be in labor, did you seek any other maternities for your delivery (“peregrination”)?”, 2.1. “How many maternities did you seek before being admitted to this institution?” and 2.2. “What was the justification of the other maternities to refuse your hospitalization for childbirth?”. The variables studied for the analysis of the factors associated with antepartum peregrination were: sociodemographic characteristics (place of residence, age range, skin color, education level, paid job and marital status), characteristics of the prenatal and childbirth care received (prenatal coverage, early initiation, number of appointments, type of service, follow-up by the same professional(s), instruction on the referral maternity for childbirth and type of funding for parturition) and characteristics of the gestational process (gestational age, number of pregnancies, number of deliveries, planned pregnancy, feelings about pregnancy and attempted abortion).

The age group’s determination followed WHO’s convention, which delimits adolescence as ranging between 10 and 19 years of age.^11^ In the evaluation of gestational age (GA), the pregnant women were sorted into two large groups: adequate GA and inadequate GA. Adequate GA was considered as ranging between 37 and 42 weeks of gestation (full term), and inadequate GA was considered as being ≤ 36 weeks and six days (preterm) or > 42 weeks (late term)^12^.

The statistical analysis was performed on the IBM^®^ Statistical Package for the Social Sciences 20.0 Mac (SPSS 20.0 Mac, SPSS Inc., Chicago, Illinois, USA). Univariate and bivariate techniques were initially used to obtain the distribution of the absolute and relative frequencies. The associations were investigated using the chi-square test between categorical variables and Fisher’s exact test for categories with low-frequency cells. The odds ratio (OR) and its respective 95% confidence intervals (95%CI) were estimated as association measure, using the Mantel-Haenszel method. In all cases, 5% significance was adopted.

Finally, the multivariate statistical analysis was carried out, with the dependent variable (outcome) being antepartum peregrination. The associations between antepartum peregrination and the exposure variables (mother’s paid job, type of prenatal care service, instruction on the referral maternity for childbirth) were described based on adjusted odds ratios in a generalized linear model, adopting the Bernoulli distribution (logistic regression) with robust standard errors.

This study was approved by the Research Ethics Committee of the Federal University of São Paulo, under opinion No. 453.279/2013, with the following CAAE: 22488213.4.0000.5546. All necessary measures were taken to ensure the secrecy and confidentiality of the information, according to resolution 466/2012 of the Ministry of Health’s National Health Council. The puerperal women signed the informed consent form, being ensured the right of refusal at any time, with no damage to the institutions.

## RESULTS

The 768 eligible puerperal women that were randomly selected to participate in this study were interviewed, with no losses or withdrawals during the research period. Almost all of them had undergone prenatal care (99.3%; n = 763), and 61.3% (n = 468) had been instructed on the referral maternity for childbirth. Antepartum peregrination was reported by 29.4% of them (n = 226). Most had sought care in another service before the present one (87.6%; n = 198), and 12.4% (n = 28) had sought two or three other units before this hospitalization. Regarding the justification given by the other maternities not to carry out the hospitalization of these pregnant women, it was noted that the “absence of on-call physician” was the most frequent (48.7%; n = 110) (Table 1).

**Table 1:** Descriptive results of the puerperal women’s answers to questions related to antepartum peregrination (n = 768). Sergipe, Brazil, 2015–2016.

Questions related to antepartum peregrination	n	%	Total

			n (%)
1. Did you receive prenatal care during this pregnancy?			768 (100)
Yes	763	99.3	
No	5	0.7	
1.1. During prenatal care, were you instructed about the referral maternity for childbirth?			763 (99.3)
Yes	468	61.3	
No	295	38.7	
2. Before being admitted to this institution, believing to be in labor, did you seek any other maternities for your delivery (“peregrination”)?			
No	542	70.6	768 (100)
Yes	226	29.4	
2.1. How many maternities did you seek before being admitted to this institution?			226 (29.4)
One	198	87.6	
Two	20	8.9	
Three or more	8	3.5	
2.2. What was the justification of the other maternities to refuse your hospitalization for childbirth?			
Absence of on-call physician	110	48.7	226 (29.4)
High-risk pregnancy (referred)	35	15.5	226 (29.4)
Not yet in labor	21	9.3	226 (29.4)
No beds available in the maternity ward	14	6.2	226 (29.4)
They did not justify it	5	2.2	226 (29.4)

The bivariate analysis of the maternal sociodemographic characteristics as well as those pertaining to the type of prenatal and childbirth care that were statistically associated with antepartum peregrination showed that pregnant women aged ≥ 20 years old (OR = 0.50; 95%CI 0.34–0.71), with high education level (OR = 0.42; 95%CI 0.31–0.59), a paid job (OR = 0.61, 95%CI 0.44–0.85), who had been instructed on the referral maternity for childbirth (OR = 0.53, 95%CI 0.39–0.73) and received prenatal (OR = 0.21; 95%CI 0.12–0.36) and childbirth care (OR = 0.80; 95%CI 0.76–0.83) from private healthcare services, were those who least peregrinated before childbirth. It should be noted that all those who peregrinated had used public healthcare services for childbirth (Tables 2 and 3).

**Table 2:** Associations between the mother’s sociodemographic characteristics and peregrination before childbirth (n = 768). Sergipe, Brazil, 2015–2016.

Maternal variables	Antepartum peregrination		p	OR (95%CI)	Total
	
	Yes (n = 226)	No (n = 542)			n (%)
	
	n (%)	n (%)			
Place of residence					
Big city/Capital	136 (30.6)	308 (69.4)	0.392	0.87 (0.63–1.19)	444 (57.8)
Countryside/Municipality	90 (27.8)	234 (72.2)			324 (42.2)
Age group (years)					
≤ 19	68 (41.5)	96 (58.5)	< 0.001	0.50 (0.34–0.71)	164 (21.4)
≥ 20	158 (26.2)	446 (73.8)			604 (78.6)
Skin color					
Mixed or black	188 (29.6)	447 (70.4)	0.812	1.05 (0.69–1.58)	635 (82.7)
White or yellow	38 (28.6)	95 (71.4)			133 (17.3
Education level					
Illiterate or Primary education	138 (38.8)	218 (61.2)	< 0.001	0.42 (0.31–0.59)	356 (46.4)
Secondary or higher education	88 (21.4)	324 (78.6)			412 (53.6)
Paid job					
No	144 (33.8)	282 (66.2)	0.003	0.61 (0.44–0.85)	426 (55.5)
Yes	82 (24)	260 (76)			342 (44.5)
Marital status					
Living with a partner	151 (31.1)	334 (68.9)	0.174	1.25 (0.90–1.73)	485 (63.2)
Living without a partner	75 (26.5)	208 (73.5			283 (36.8)

**Table 3:** Associations between the characteristics of prenatal care and peregrination before childbirth (n = 768). Sergipe, Brazil, 2015–2016.

Prenatal variables	Antepartum peregrination		p	OR (95%CI)	Total
	
	Yes (n = 226)	No (n = 542)			n (%)
	
	n (%)	n (%)			
Early initiation of prenatal care					
Yes	133. (30.6)	302 (69.4)	0.449	0.88 (064–1.21)	435 (57)
No	92 (28)	236 (72)			328 (43)
Number of appointments					
6 or more appointments	169 (29.6)	401 (70.4)	0.867	1.03 (0.72–1.47)	570 (74.7)
≤ 5 appointments	56 (29)	137 (71)			193 (25.3)
Type of prenatal care service					
Public	193 (35.3)	353 (64.7)	< 0.001	0.21 (0.12–0.36)	546 (71.6)
Private	16 (10.4)	138 (89.6)			154 (22)
Monitoring by the same professional(s)					
Yes	200 (29.8)	472 (70.2)	0.653	1.11 (0.68–1.82)	672 (88.1)
No	25 (27.5)	66 (72.5)			91 (11.9)
Instructed about the referral maternity for childbirth					
Yes	114 (24.4)	354 (75.6)	< 0.001	0.53 (0.39–0.73)	468 (61.3)
No	110 (37.4)	185 (62.6)			295 (38.7)
Type of funding of the place of childbirth					
Public	226 (34.2)	434 (65.8)	< 0.001	0.80 (0.76–0.83)	660 (85.9)
Private	0 (0)	108 (100)			108 (14.1)

*Fisher’s Exact Test.

The descriptive results also showed that, among the women with inadequate gestational age (13.4%; n = 103), in their first pregnancy (43.2%; n = 332), with no other children (4.8%; n = 37), dissatisfied with pregnancy (35.9%; n = 276) and who reported abortion attempts during the prenatal follow-up (4.9%; n = 38), most of them peregrinated before childbirth. However, no statistical evidence of association between any of these variables was observed (p > 0.05) (Table 4).

**Table 4:** Associations between the characteristics of the gestational process and peregrination before childbirth (n = 768). Sergipe, Brazil, 2015–2016.

Maternal variables	Antepartum peregrination		p	OR (95%CI)	Total
	
	Yes (n = 226)	No (n = 542)			n (%)
	
	n (%)	n (%)			
Gestational age					
Adequate (37 to 42 weeks)	191 (28.7)	474 (71.3)	0.296	1.27 (0.82–1.98)	665 (86.6)
Inadequate (≤ 36 and ≥ 43 weeks)	35 (34)	68 (66)			103 (13.4)
Number of pregnancies					
First pregnancy	108 (32.5)	224 (67.5)	0.100	0.77 (0.56–1.05)	332 (43.2)
More than one pregnancy	118 (27.1)	318 (72.9)			436 (56.8)
Number of childbirths					
First child	12 (32.4)	25 (67.6)	0.712	0.86 (0.42–1.74)	37 (4.8)
More than one child	214 (29.3)	517 (70.7)			731 (95.2)
Planned pregnancy					
Yes	93 (29.4)	223 (70.6)	0.999	1.00 (0.72–1.37	316 (41.1)
No	133 (29.4)	319 (70.6)			452 (58.9)
Feelings about pregnancy					
Satisfied	137 (27.8)	355 (72.2)	0.199	1.23 (0.89–1.69)	492 (64.1)
Unsatisfied	89 (32.2)	187 (67.8)			276 (35.9)
Attempted abortion					
Yes	16 (42.1)	22 (57.9)	0.099	1.80 (0.92–3.49)	38 (4.9)
No	210 (28.8)	520 (71.2)			730 (95.1)

*Fisher’s Exact Test.

In the multivariate analysis, after adjustments for confusion variables, it was noted that antepartum peregrination was in fact less frequent among women with a paid job (adjusted OR = 0.18; 95%CI 0.41–0.82), who had been instructed during prenatal care about the referral maternity for childbirth (adjusted OR = 0.88; 95%CI 0.42–0.92), and who used private healthcare services to receive prenatal (adjusted OR = 0.44; 95%CI 0.18–0.86) or childbirth (adjusted OR = 0.96; 95%CI 0.66–0.98) care.

**Table 5:** Adjusted odds ratio for variables determining peregrination before childbirth (n = 226). Sergipe, Brazil, 2015–2016.

Independent variables	Dependent variable: antepartum peregrination

	OR_a_ (95%CI)
Mother with paid job	
Yes	0.59 (0.41–0.82)
No	1
Type of prenatal care service	
Private	0.44 (0.18–0.86)
Public	1
Instruction about the referral maternity for childbirth	
Yes	0.88 (0.42–0.92)
No	1
Type of financing used for childbirth	
Private	0.96 (0.66–0.98)
Public	1

## DISCUSSION

The present study showed the antepartum peregrination rate to be 29.4% among women from Sergipe, a result higher than that found in Brazil (16.2%) and in the Northeast region in general (25.1%)^4^, considering the interference of the mother’s socioeconomic factors, type of prenatal care and source of funding for childbirth in the occurrence of this phenomenon.

It was shown that the prenatal care received by the women failed to comply with the recommendations of the Program of Humanization of Prenatal Care and Childbirth (PHPN)^13^, especially with regard to their connection to the referral maternity for childbirth, since only a little more than half of the women interviewed were instructed on it during follow-up. This lack of compliance was also found in other national studies with a similar design, which found instruction percentages lower than 60%^4,14,15^.

Authors argue that one of the objectives of the early connection to the referral maternity is the reduction of peregrination at the time of labor^14^ The present study confirmed the higher frequency of peregrination when the women were not instructed on it during prenatal care.

We also identified that the majority of the parturients who peregrinated before childbirth sought care in a single service before the present one, similarly to the finding of another Brazilian state-wide study, in which 70.7% of the 2,228 patients evaluated were admitted to the second maternity visited^1^. The main justification given by the other maternities not to accept the hospitalization of the parturients of the present study was the “absence of on-call physician” at the time of their arrival at the health service. This raises questions about the autonomy of obstetrician nurses in the maternity wards of Sergipe, since these professionals also have legal support to assist normal-risk vaginal deliveries throughout the national territory. It is supposed that, in health care practice, although resolution 516/2016 of the Federal Nursing Council ensures obstetricians and obstetric nurses the right of issuing hospital admission authorization reports (AIH) for normal delivery procedures without dystocia, as well as of performing the obstetric follow-up of the women and newborns under their care, from the moment of their hospitalization until discharge^16^, many childbirth institutions in the country still leave these assignments under the responsibility of physicians only, with the possible legal argumentation that these professionals are responsible for dealing with serious events that may result in maternal or fetal death.

Moreover, this result differs from other studies, which identified the lack of beds available in the institution^1,17^ and high-risk pregnancy^18^ as the main causes of antepartum peregrination. It is pointed out that, regardless of the peregrination’s etiological factor, this phenomenon contributes to the excessive delay in childbirth care, which can lead to complications in parturition that often evolve to maternal or fetal death^19,20^.

In this context, it is understood that the knowledge of the maternal characteristics associated with antepartum peregrination may support the development of effective strategies to connect these pregnant women to the referral maternity for childbirth. Older parturients, with high education level and a paid job were those who showed the lowest peregrination rates. It is believed that the lower rates among women of higher socioeconomic status are related to the possibility of access to private healthcare services for parturition, where peregrination is certainly less frequent due to the fact of these services being paid.

Adolescence and low education level were also indicated by other authors as maternal variables that are predictive of this problem^1,17,21^. It is known that teenage pregnancy increases the risk of obstetric complications, with the possibility of negative repercussions for mother and newborn, also being associated with psychosocial and economic problems related to the early constitution of motherhood^22^. Thus, not connecting these adolescents to the referral maternity for childbirth during prenatal care may be considered an important aggravating factor that requires the provision of emergency care by the health professionals responsible for this follow-up.

It should be noted that inadequate prenatal care is one of the main causes of antepartum peregrination^1,5,7,8^. Many studies have identified prenatal care failures that are capable of interfering in its quality and effectiveness, such as low coverage^23^, late initiation, inadequate distribution of appointments^24^, incomplete performance of the recommended procedures^25^, and, with even greater relevance for the issue of antepartum peregrination, lack of information about the referral maternity for childbirth during this process^4^.

The influence of the parturient women’s gestational and obstetric characteristics in the access to the referral maternity for childbirth is also mentioned^18^. The descriptive results of the present study showed that the majority of the parturients who peregrinated were women with inadequate gestational age, in their first pregnancy, with no other children, dissatisfied with pregnancy and who reported abortion attempts during pregnancy.

Gestational age at birth is the main predictor of neonatal health^26^. In Brazil, as in other countries, there has been a constant reduction in this GA, with each time more babies being born in the late preterm and early term ranges compared to previous years^27^. This situation may also motivate the refusal of admission for childbirth by the first service sought by the pregnant woman, with consequent referral or peregrination to medium and high-complexity maternities^18^.

Antepartum peregrination becomes even more conflicting when considering that most patients seek care in another institution by their own means and, commonly, using means of transport with inadequate comfort and safety^1,17^. A study conducted in the city of Rio de Janeiro showed that only one fifth of the women had been transported by an ambulance during labor^1^.

The limitations of this study are related to the reliability of the data obtained from the reports of the puerperal women interviewed, such as whether they were instructed on the referral maternity for childbirth during prenatal care, the number of maternities visited before hospitalization for labor and the justification of these other maternities not to accept their hospitalization.

In this context, it should be noted that high antepartum peregrination rates were found in Sergipe, with interference of the mother’s socioeconomic factors, the characteristics of the prenatal care received and the type of financing for childbirth. Antepartum peregrination was less frequent among women aged ≥ 20 years old, with high education level and a paid job, who had been instructed during prenatal care about the referral maternity for childbirth, and who used private healthcare services to receive prenatal or childbirth care. No statistical evidence of associations between gestational characteristics and the occurrence of peregrination was observed.

The need for greater commitment on the part of the health professionals responsible for prenatal care was noted, since the early connection of the pregnant women to the referral maternity for childbirth is essential for the prevention of peregrination and consequent reduction in the rate of maternal and fetal risks related to parturition. It is also suggested that municipal, state and federal managers pay greater attention to the organization of maternal and child health services in the country, ensuring that pregnant women have access to timely, safe and humanized childbirth, as well as the appropriate fulfilment of their sexual and reproductive rights.
